# Single-cell atlas of photoaged skin reveals JAK-STAT blockade as a strategy to reverse dermal remodeling

**DOI:** 10.3389/fimmu.2026.1748123

**Published:** 2026-07-01

**Authors:** Mingwang Zhang, Yuanyuan Liu, Cheng Yang, Chenhui Wang, Yurui Zou, Kai Luo, Haima Li, Rongya Yang

**Affiliations:** 1 Department of Dermatology, Southwest Hospital, Army Medical University, Chongqing, China; 2 Chinese People’s Liberation Army (PLA) Medical School, Beijing, China; 3 Department of Dermatology, Seventh Medical Center of Chinese People’s Liberation Army (PLA) General Hospital, Beijing, China; 4 Reahealth Inc., Beijing, China; 5 Bioinformatics Center, Academy of Military Medical Sciences (AMMS), Beijing, China; 6 The 83rd Affiliated Hospital, Xinxiang Medical University, Xinxiang, Henan, China; 7 Biological Therapy Center, Seventh Medical Center of Chinese People’s Liberation Army (PLA) General Hospital, Beijing, China; 8 Department of Neurosurgery, No. 215 Hospital of Shaanxi Nuclear Industry, Xianyang, Shaanxi, China

**Keywords:** fibroblast, JAK-STAT signaling, ruxolitinib (JAK1/2 inhibitor), SASP, single-cell RNA sequencing (scRNA-seq), skin photoaging, ultraviolet (UVA/UVB)

## Abstract

**Introduction:**

Chronic ultraviolet (UV) exposure superimposes inflammatory and matrix-degenerative insults on intrinsic aging to produce cutaneous photoaging, yet the cell-type-resolved circuits and druggable nodes driving this process remain poorly defined.

**Methods:**

Chronic We established a chronic UVA+UVB-induced mouse model of photoaging and performed single-cell RNA sequencing of control, UV-exposed, and UV + ruxolitinib-treated dorsal skin to map photoaging and therapeutic response at cellular resolution.

**Results:**

Chronic Across 14 major lineages, UV exposure preserved overall cellular composition but induced a coordinated transcriptional network shift, marked by activation of JAK–STAT/IRF/AP-1-driven inflammatory and oxidative-stress programs and suppression of developmental, DNA-repair, and differentiation modules. Fibroblast reclustering resolved eight states and identified a papillary-to-reticular transitional continuum with the highest transcriptional burden, integrating senescence-associated secretory phenotype and extracellular-matrix disassembly signatures and skewing pseudotime toward late, terminal states. Topical JAK1/2 inhibition with ruxolitinib attenuated clinical and histological photoaging phenotypes, restored collagen and elastic fiber architecture, and rebalanced fibroblast trajectories by suppressing inflammatory/stress pathways while reactivating basement-membrane, extracellular-matrix, adhesion, and wound-healing programs. Cross-lineage analysis further revealed reversal of conserved stress–metabolic axes, including *Hmox1*, *Gpx3*, *Ucp2*, and *Nr4a2*, restoration of structural–matrix programs involving Dcn, Gsn, and Nup210l, normalization of aging regulators such as *Cdkn1a* and *Trp53*, and re-establishment of dermal–epidermal crosstalk through collagen IV–syndecan and fibronectin/laminin–CD44/αvβ1 signaling.

**Discussion:**

Chronic These findings identify transitional fibroblasts as central executors of UV-driven dermal remodeling and support JAK1/2 inhibition as a network-level strategy to restore dermal–epidermal homeostasis in photoaged skin.

## Introduction

Skin, the body’s largest barrier organ, undergoes multifactorial decline with age, coupling intrinsic programs (stem-cell exhaustion, genomic instability, and epigenetic alterations) to lifelong environmental insults (1, 2). Among these, chronic ultraviolet (UV) exposure is dominant: it superimposes persistent, low-grade inflammation and matrix degeneration on intrinsic aging to yield the clinical syndrome of photoaging, including wrinkling, laxity, dryness, desquamation, and impaired repair (2–4). While these hallmarks are well recognized, how distinct cutaneous cell types and their interactions encode photoaging, and which nodes are tractable to intervention, remains unresolved because bulk assays blur cellular heterogeneity and obscure cross-layer communication. Single-cell RNA sequencing (scRNA-seq) overcomes these limitations by resolving cell-type programs, lineage dynamics, and intercellular signaling within native tissue (5, 6). Yet causal dissection in humans is constrained by heterogeneous UV exposures and limited experimental control. A chronic UVA+UVB induced photoaging mouse model therefore provides a controlled platform to map the cellular architecture of photoaging and to test targeted interventions at single-cell resolution.

Here, we combined a photoaging model with scRNA-seq to chart the photoaged skin ecosystem. We identified 14 major cell classes and resolved eight fibroblast (FB) subpopulations, revealing cross-lineage reprogramming characterized by up-regulated inflammatory/oxidative-stress modules and broad suppression of developmental and differentiation programs. Transitional fibroblasts (FB2–FB3) emerged as a papillary-to-reticular bridge that amplified JAK-STAT/IRF/AP-1 signaling, whereas papillary subsets showed widespread functional suppression, implicating a layered response in which the papillary dermis is first debilitated and transitional FBs then drive matrix breakdown and reticular remodeling.

Motivated by the prominence of JAK-STAT activity across fibroblasts and keratinocytes, we tested topical JAK1/2 inhibition (ruxolitinib) as a network-level perturbation. At the tissue level, ruxolitinib attenuated epidermal hyperplasia, dermal inflammation, and senescent-cell burden. At the single-cell level, it produced a directional rescue: dampening JAK-STAT/IRF/AP-1 modules and stress/senescence-associated secretory phenotype (SASP) signatures while restoring extracellular-matrix (ECM), dermal–epidermal junction (DEJ), adhesion, and wound-healing programs, with the strongest effects concentrated in FB2–FB3. Ligand–receptor analysis further indicated re-established dermal–epidermal crosstalk (e.g., collagen IV–syndecan), mechanistically linking transcription-factor rewiring to DEJ stabilization and collagen reorganization. Collectively, our study closes the loop between a disease driver (the JAK-STAT axis) and an actionable intervention (JAK1/2 blockade), localizes efficacy to transitional fibroblasts, and outlines a cell-resolved framework for anti-photoaging therapy centered on inflammation control and ECM/DEJ reconstruction.

## Results

### Single-cell profiling identifies 14 skin cell types and structural changes in photoaged mouse skin

**Figure 1:**
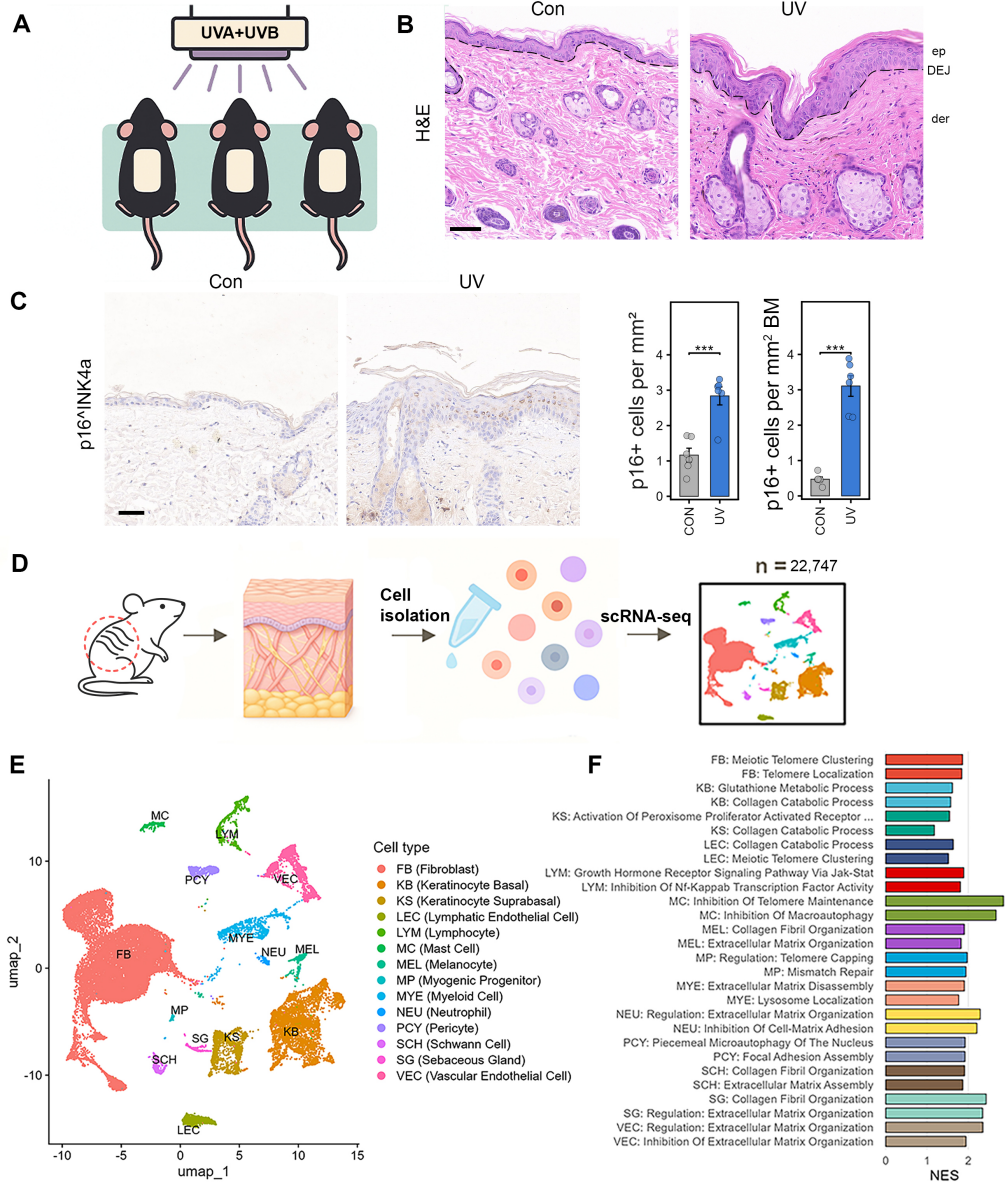
Cellular taxonomy and tissue remodeling in murine photoaging. **(A)** Experimental paradigm for chronic UV exposure to induce cutaneous photoaging in mice. **(B)** Hematoxylin and eosin staining of dorsal skin. Scale bar, 50 µm. **(C)** Left: p16^INK4a staining. Scale bar, 50 µm. Right: quantitative morphometry: p16^+ cells per mm² dermis, and p16^+ cells per mm basement membrane (BM). Statistics as in **(B)**. **(D)** Single-cell RNA-seq workflow. Murine dorsal skin was enzymatically dissociated; captured single cells were profiled by scRNA-seq and processed through a standardized bioinformatics pipeline. **(E)** UMAP embedding resolving 14 transcriptionally distinct clusters corresponding to major skin lineages: fibroblast (FB), basal keratinocyte (KB), suprabasal keratinocyte (KS), lymphatic endothelial cell (LEC), vascular endothelial cell (VEC), pericyte (PCY), sebaceous gland (SG), melanocyte (MEL), mast cell (MC), lymphocyte (LYM), myeloid cell (MYE), Schwann cell (SCH), myogenic progenitor (MP), and neutrophil (NEU). **(F)** Top Gene Ontology Biological Process (GO-BP) enriched in UV group per cell type.

We established a chronic UVA+UVB mouse model of cutaneous photoaging (**Figure 1A** and **Supplementary Figure 1A**). Repeated irradiation elicited overt photoaging in dorsal skin, including coarse texture, wrinkling, dryness, mottled hyperpigmentation, and reduced recoil (**Supplementary Figure 1B**). Histologically, UV exposure produced epidermal thickening with compact hyperkeratosis and occasional “sunburn cells” (apoptotic suprabasal keratinocytes), as previously reported (7). The dermal collagen network was disorganized and fragmented, replacing the fine fibrillar architecture seen in controls. The upper dermis showed prominent neutrophil and lymphocyte infiltration (**Figure 1B**, **Supplementary Figure 1C**) (8). Senescent cells accumulated with UV irradiation: p16^INK4a^+ cells increased in both epidermis and dermis, frequently adjacent to fragmented collagen and just beneath the DEJ (**Figure 1C**). qRT-PCR confirmed the UV irradiation-triggered damage and inflammation: Il6, Il1b and H2ax were increased, whereas Tgfb1 decreased (**Supplementary Figure 1D**). The above results indicate that UV-induced DNA-damage responses and cell-cycle suppression extend from the epidermis into the superficial dermis, with concurrent inflammatory activation, consistent with the manifestations of photoaged skin.

To resolve cell-type-specific changes underlying these phenotypes, we performed scRNA-seq on control and UV-exposed skin. After rigorous quality control, 22,747 cells were retained (**Figure 1D**). Uniform Manifold Approximation and Projection (UMAP) revealed 14 robust clusters corresponding to major cutaneous lineages, annotated by canonical markers: fibroblast (FB; Col1a1, Col3a1), basal keratinocyte (KB; Krt14, Krt5), suprabasal keratinocyte (KS; Krt1, Krt10), lymphatic endothelial cell (LEC; Lyve1, Prox1), lymphocyte (LYM; Cd3d, Cd3e), mast cell (MC; Cpa3, Kit), melanocyte (MEL; Tyr, Dct), myeloid cell (MYE; Lyz2, C1qa), myogenic progenitor (MP; Pax7, Myod1), neutrophil (NEU; S100a8, S100a9), pericyte (PCY; Pdgfrb, Acta2), Schwann cell (SCH; Plp1, Mpz), sebaceous gland (SG; Scd1, Cidea), and vascular endothelial cell (VEC; Pecam1, Cdh5) (**Figure 1E**, **Supplementary Figures 1E–G**).

Notably, cellular composition was broadly preserved with photoaging: all 14 lineages were present in both groups, and their relative abundances remained comparable (**Supplementary Figure 1H**). Thus, in this model, chronic UV does not extinguish or create major cell classes; however, it may rewire the functional programs within existing populations. UV exposure enriched ECM-catabolic and adhesion/organization programs across keratinocytes, fibroblasts, endothelium, and pericytes-coherent with canonical matrix metalloproteinase (MMP)-driven matrix breakdown that underpins dermal photoaging (**Figure 1F**). Concurrently, immune/stromal compartments tilted toward inflammatory JAK-STAT and lysosome-autophagy biology, while parenchymal lineages engaged antioxidant and genome-maintenance responses, in line with established UV-induced oxidative and DNA-damage pathways.

### Gene expression dynamics of photoaging at single-cell resolution

**Figure 2:**
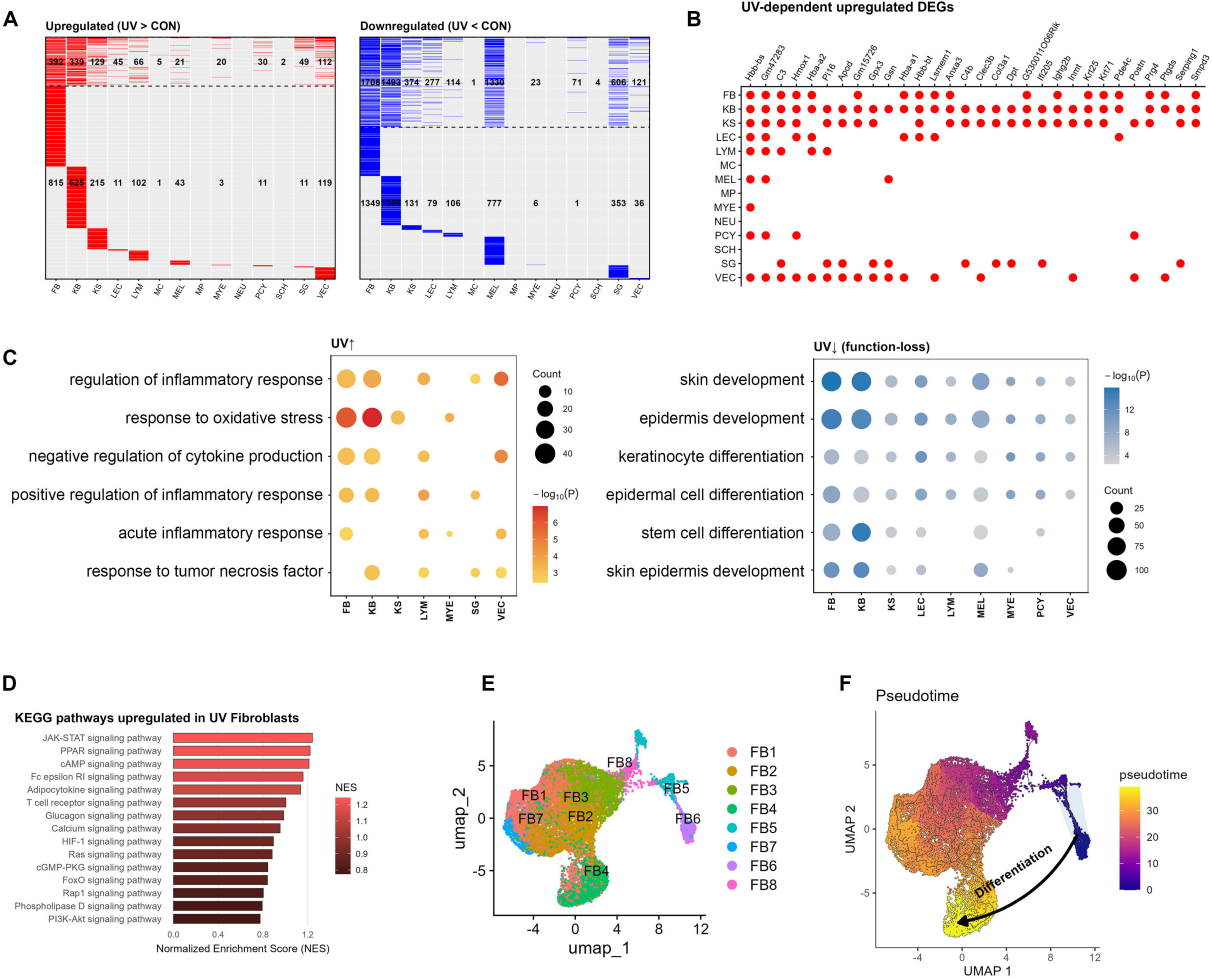
Systems-level remodeling of lineage programs and fibroblast state dynamics in murine photoaging. **(A)** Global landscape of differential expressions across skin lineages after UV exposure. Heatmap partitions UV-induced differentially expressed genes (DEGs) into up-regulated (left, red) and down-regulated (right, blue) modules for each cell type (columns); grey, non-significant. The upper tier (above dotted line) denotes DEGs shared by ≥2 lineages, whereas the lower tier captures lineage-restricted changes. **(B)** Cross-lineage convergence of photoaging signatures. Dot plots highlight representative shared DEGs. UV-upregulated genes present in ≥3 lineages. **(C)** Lineage-resolved functional enrichment. Dot plots summarize GO-BP terms associated with UV-responsive programs across cell types; left, terms derived from up-regulated DEGs; right, terms from down-regulated DEGs. Dot size reflects DEG counts; color intensity reflects enrichment *p*-value. **(D)** Bar plot of fibroblast-centric KEGG pathway activation. **(E)** UMAP plot of fibroblasts subtypes. **(F)** State transitions along fibroblast trajectories. Pseudotime ordering delineates progressive shifts in fibroblast states, with cells colored by inferred pseudotime.

To delineate cell type-specific transcriptional reprogramming in photoaged skin, we identified differentially expressed genes (DEGs; logFC > 0.25; p adj < 0.05) between UV-exposed and control samples across all major skin lineages (**Figure 2A, B**; **Supplementary Figure 2A**). Fibroblasts exhibited the most extensive perturbation, with 1,207 genes up-regulated and 3,055 genes down-regulated in UV (total 4,262), underscoring fibroblasts as the principal responders to cutaneous photoaging. Notably, 67.5% of fibroblast UV-up genes were fibroblast-specific, consistent with directional activation of a focused stress/inflammatory program, whereas 55.9% of UV-down genes were shared across cell types, indicating convergent, cross-lineage repression (**Figure 2A, B**; **Supplementary Figure 2A**). Pathway enrichment further revealed a coherent skin-wide signature, with inflammation and oxidative stress up-regulated, tissue development/differentiation broadly repressed, and fibroblasts and keratinocytes as the dominant effector compartments (**Figure 2C**). Concordantly, DNA damage-repair genes were significantly reduced in fibroblasts and basal keratinocytes in photoaged skin (**Supplementary Figure 2B**) (**Table 1**), aligning with the canonical photoaging axis of functional decline coupled to chronic, low-grade inflammation (inflammaging).

**Table 1:** Curated marker and functional gene sets used for fibroblast subset annotation and pathway/module analyses. Papillary and reticular fibroblast marker genes were used to assign dermal fibroblast identities, while the DNA damage/repair and senescence-associated gene set was used to evaluate UV-induced cellular stress, inflammatory activation, oxidative stress, apoptosis, cell-cycle regulation, and telomere-related responses.

papillary genes	Dpp4, Lef1, Efemp1, Wif1, Col6a6, Col18a1, Crabp1
reticular genes	Mfap5, Lox, Sfrp4, Col11a1, Comp, Dcn
DNA repair	Cdkn1a, Cdkn2a, P53, Gadd45a, Rad51, Xpc, Il6, Il1b, Tnf, Tgfb1, Ccl2, Mmp9, Mmp13, Sod1, Sod2, Nrf2, Hmox1, Prdx1, Bax, Bcl2, Casp3, Casp9, Puma, Fas, Fasl, Cdkn1a, Cdkn2a, CyclinD1, Rb1, Cdk4, Tert, Terc, Ttelomere, Trf1

As aging elevates transcriptional noise, we quantified cell type-wise coefficients of variation (CV) (9). Fibroblasts displayed the highest CV, indicating amplified expression heterogeneity (**Supplementary Figure 2C**). Using a predefined SASP panel, we assessed within-lineage DEG composition and found the broadest SASP up-regulation in fibroblasts and followed by keratinocytes (**Supplementary Figure 2D**). Across DEG burden, Gene Ontology Biological Processes (GO-BP), CV, and SASP breadth, fibroblasts consistently emerged as the most profoundly affected cell type in UV-induced aging. Moreover, in fibroblasts specifically, GO-BP analysis highlighted directional activation of inflammation/stress pathways, most prominently JAK-STAT, implicating this axis as a central driver of inflammaging and matrix remodeling (**Figure 2D**).

Dermal fibroblasts sustain lifelong ECM synthesis and remodeling, thereby establishing dermal architecture and mechanics; through spatial and lineage diversification into papillary versus reticular identities, they also maintain the epidermal and hair-follicle niches (10). How discrete fibroblast subsets differ in susceptibility and plasticity during photoaging remains incompletely resolved (11). UMAP partitioned fibroblasts into eight subtypes (FB1-FB8) (**Figure 2E**). Marker expression placed FB1-FB4 as reticular-like and FB5/FB6/FB7 as papillary-like (**Supplementary Figure 2E**; **Table 1**). SASP genes (Il6, Il1b) shifted from sparse/zero-inflated in controls to broad, high-amplitude expression in transitional/reticular-leaning FB2→FB3 and FB1/FB4; chemokines (Ccl2, Cxcl1/Cxcl2) and matrix-effectors (Mmp3, Mmp13, Serpine1/Timp1) followed the same pattern, whereas papillary-like FB5/FB6 showed only modest right-shifts (**Supplementary Figure 2F**). UV further induced stress/inflammation and adhesion-remodeling nodes (JAK-STAT and oxidative stress markers; Cd44, Adam19; innate/IFN-linked genes Ifi27l2a/Ifitm1; and glycolytic Hk2) (**Supplementary Figure 2G**). Subtype-resolved heatmaps localized UV-upregulation to reticular-like FB1-FB4, with the strongest transcriptional suppression in papillary FB6 (**Supplementary Figures 2H** and **2I**). FB2 and FB3 carried the largest total DEG loads and most uniquely up-regulated genes under UV, whereas FB6 underwent broad attenuation. Pseudotime reconstructed a papillary-to-reticular continuum and positioned FB2 (earlier) and FB3 (later) as transitional nodes (**Figure 2F**), with positive correlation between senescence scores and pseudotime coordinates (**Supplementary Figure 2J**); oxidative-stress, inflammatory signaling, and JAK-STAT3 were specifically enriched in FB2/FB3 (**Supplementary Figure 2K**). These data support a hierarchical model in which UV first suppresses papillary programs (FB5/FB6) and then amplifies inflammation/ECM disassembly in transitional FB2/FB3, driving maladaptive remodeling within the reticular dermis-mechanistically consistent with known papillary/reticular lineage roles.

### Topical JAK1/2 inhibition alleviates photoaging phenotypes in mice

**Figure 3:**
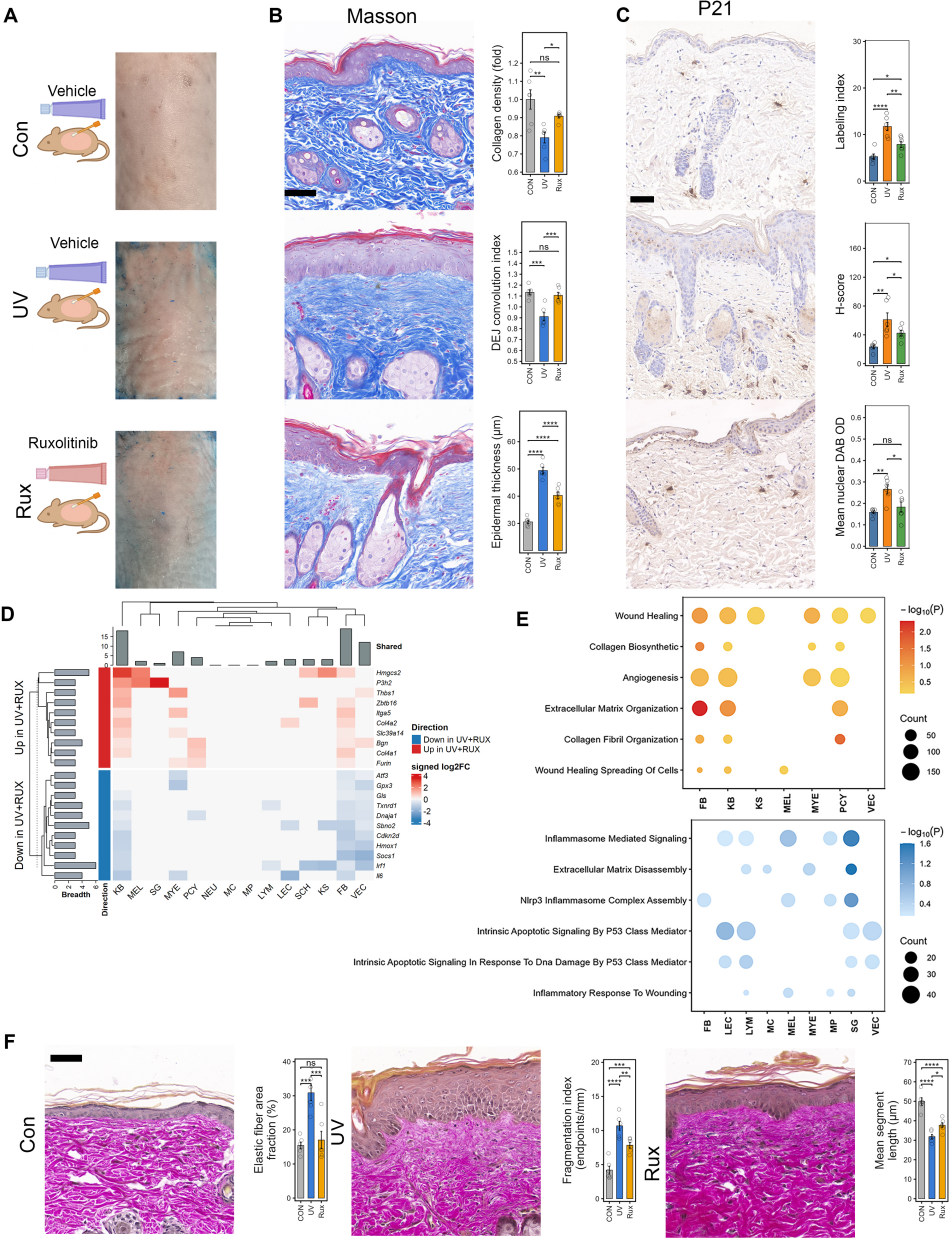
Ruxolitinib partially restores tissue architecture and transcriptional programs in UV-photoaged skin. **(A)** Experimental design and representative phenotypes. Schematic of three groups-Control (CON; no UV, vehicle), UV + Vehicle (UV), and UV + Ruxolitinib (Rux)-with dorsal skin images. **(B)** Representative Masson’s trichrome. Scale bar, 50 µm. Quantification bar plots. Collagen density, DEJ convolution index, and Epidermal thickness. (n = 6). Bars show mean ± SE; ns ≥ 0.05; * < 0.05; ** < 0.01; *** < 0.001; **** < 0.0001. **(C)** Representative p21^Cip1^ immunohistochemistry. Scale bar, 50 µm. Quantification bar plots. Labeling index, H-score, and mean nuclear DAB optical density. Statistics as in **(B)**. **(D)** Heatmaps of genes differentially expressed between Rux group and UV group: up-regulated (red); down-regulated (blue). Genes shown are differentially expressed in at least three major cell types. **(E)** Dot plot of GO biological process enrichment in Rux compared to UV; dot size denotes the number of associated counts per term. Top: upregulated in Rux group. Bottom: downregulated in Rux group. **(F)** Representative Elastica-van Gieson (EVG) micrographs illustrating fiber architecture. Quantification: elastic fiber area fraction, fragmentation index, and mean segment length. Statistics as in **(B)**.

Building on prior evidence that JAK2 inhibition suppresses SASP factor secretion in UVB-irradiated cells and that topical ruxolitinib attenuates JAK-STAT-driven Th2/IFN inflammation in murine dermatitis, we posited that JAK1/2 blockade would mitigate or partially reverse the histologic and molecular hallmarks of cutaneous photoaging (12–14). Following Mor et al., UV-exposed mice were treated topically with ruxolitinib and compared with UV + vehicle controls (**Figure 3A**) (15). Ruxolitinib yielded a visibly milder photoaging phenotype: wrinkle onset was delayed, wrinkle number and depth were reduced, dryness and desquamation involved smaller areas. Histologically, ruxolitinib-treated skin displayed diminished epidermal hyperplasia and keratinization abnormalities, reduced dermal inflammatory-cell density, and a narrowed perivascular/peri adnexal mononuclear infiltrate (**Figure 3B**). The UV-induced accumulation of p21-positive cells, indicative of DNA-damage responses and cell-cycle arrest, was markedly reduced in the epidermis and superficial dermis, with a particularly notable decrease among papillary-dermal fibroblasts adjacent to fragmented collagen (**Figure 3C**). In keeping with chronic UV-induced growth arrest, the UV regimen reduced the basal-layer Ki-67 index, whereas ruxolitinib partially restored proliferative tone toward control without overshoot (**Supplementary Figures 3A, B**), consistent with published chronic-UV mouse and human photoaging data. Consistently, Il6, Il1b, H2afx, and Tgfb1 mRNA levels measured by qPCR were restored (**Supplementary Figure 3C**). Collectively, these data demonstrate that topical ruxolitinib substantially ameliorates the clinical and histopathological features of photoaging, indicating a partial rescue of cutaneous homeostasis.

To define the cellular and molecular basis of these effects, we performed scRNA-seq on skin from UV + ruxolitinib-treated mice. UMAP visualization revealed no emergent outlier clusters, and overall cell-type composition in the ruxolitinib group was comparable to UV and control (**Supplementary Figure 3D**), indicating that cell identities were not overtly altered by treatment. A heatmap of per-cell-type DEG counts (**Supplementary Figure 3E**) confirmed that fibroblasts harbored the greatest transcriptional shifts in Rux vs UV, underscoring fibroblasts as primary targets of intervention. Cataloguing DEGs within each lineage showed that transcripts linked to interferon/inflammatory or oxidative-stress responses were robustly down-regulated by ruxolitinib, whereas genes involved in basement membrane, extracellular matrix, and adhesion were reciprocally up-regulated. In fibroblasts, and similarly in basal keratinocytes, ruxolitinib reduced Irf1, Il6, Socs1, Atf3, Gpx3, and Txnrd1, while increasing Col4a1, Col4a2 (type IV collagen; key DEJ component), Thbs1, Itga5, Bgn, P3h2, and Zbtb16 (**Figure 3D**). These concerted shifts point to a cross-lineage realignment of the core photoaging network.

Pathway-level analyses reinforced this transition from inflammation to repair. Gene-set enrichment of Rux group showed significant gains in wound-healing programs, collagen fibril organization, and extracellular-matrix assembly across fibroblasts, basal keratinocytes, and suprabasal keratinocytes, accompanied by reduced enrichment of inflammasome-related signaling (including NLRP3 complex assembly), ECM disassembly, p53-mediated intrinsic apoptosis/DNA-damage response, and wound inflammatory response (**Figure 3E**). Collagen rescue was corroborated by Elastica-van Gieson (EVG) staining (**Figure 3F**). Thus, JAK1/2 inhibition reprograms photoaged skin from a pro-inflammatory, degradative state toward matrix remodeling and tissue repair, with coordinated effects along the epidermal-dermal-vascular axis that aligns with restoration of DEJ/ECM architecture.

To quantify “rescue,” we intersected DEGs from the UV-Control contrast with those from Rux-UV; genes whose UV-induced changes were significantly reversed by ruxolitinib were designated rescued (**Supplementary Figure 3G**). Two cross-lineage axes emerged. First, a stress-metabolic axis (Hmox1, Gpx3, Ucp2, Nr4a2, Per1, Plgds) was prominently normalized in fibroblasts and keratinocytes and extended to myeloid, vascular endothelial, and sebaceous lineages, indicating reversal of UV-triggered inflammatory/oxidative imbalance. Second, a structural-matrix axis (Dcn, Gsn, Nup210l) was concomitantly restored across multiple lineages, pointing to re-established ECM and cytoskeletal homeostasis. Linking these rescues to canonical aging nodes, cross-reference with GenAge revealed consistent reversal of key stress/metabolic and aging regulators (Gdf15, Mif, Ucp2, Cdkn1a (p21), Trp53, Txn1, Clock, and Insr) across fibroblasts, keratinocytes, and additional lineages (**Supplementary Figure 3F**). Concordantly, pathway enrichment showed down-shifts in damage/stress and senescence programs (e.g., nucleotide excision repair/base excision repair, double-strand break repair, oxidative-stress-linked intrinsic apoptosis, replicative senescence) and enhanced collagen biosynthetic pathways, mirroring histomorphology improvements (**Supplementary Figure 3H**). Together, these findings establish that ruxolitinib reverses UV-imposed transcriptional programs and redirects the network from pro-inflammatory/damage toward repair/remodeling.

### Ruxolitinib rejuvenates transitional fibroblasts and restores dermal-epidermal homeostasis

**Figure 4:**
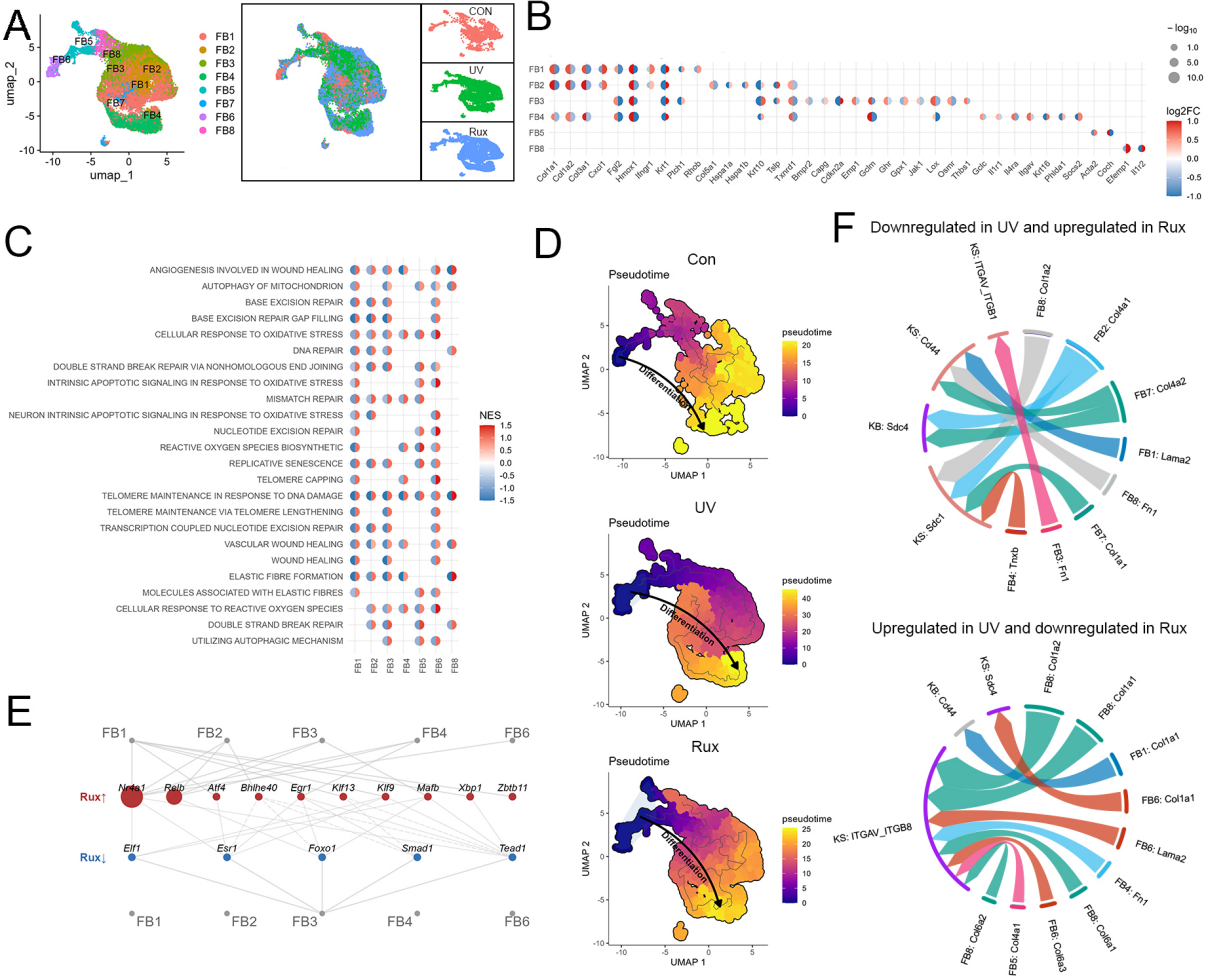
Ruxolitinib reprograms fibroblast states, lineage trajectories, and intercellular signaling in UV-photoaged skin. **(A)** Fibroblast embedding across conditions. UMAPs of all fibroblasts colored by cell subset **(left)** and by treatment (right; CON, UV, Rux). **(B)** Gene-level rescue of photoaging signatures. Dot plot of ageing-associated DEGs in fibroblasts whose UV-induced change is directionally reversed by ruxolitinib. Left semicircle encodes CON vs UV (red, up in UV; blue, down in UV); right semicircle encodes UV vs Rux (red, increased with Rux; blue, decreased with Rux). Dot size reflects statistical significance (adjusted *p*). **(C)** Pathway-level rescue in fibroblasts. Dot plot of GO Biological Process terms whose enrichment direction in CON vs UV is inverted by ruxolitinib in UV vs Rux. Semicircle color coding as in **(B)**. **(D)** Pseudotime projection of fibroblasts colored by inferred pseudotime highlights UV-biased progression toward late states and partial normalization with ruxolitinib. **(E)** Regulatory network of rescued transcription factors by Rux. **(F)** Cell-cell communication rescue. Circos diagram of ligand-receptor pairs between fibroblasts and keratinocyte compartments (KB, basal; KS, suprabasal) that are perturbed by UV and shifted toward CON levels by ruxolitinib; link thickness denotes interaction strength.

Fibroblasts are the principal cellular arbiters of photoaging; we therefore examined how JAK1/2 blockades reshape fibroblast-state dynamics. At the level of cluster composition, ruxolitinib preserved the overall fibroblast taxonomy observed in control and UV-exposed skin (**Figure 4A**). Functionally, however, the inhibitor broadly counteracted UV-driven transcriptional imbalances across fibroblast subtypes, augmenting ECM/collagen programs (Col1a1, Col1a2, Col3a1) while suppressing inflammatory and oxidative-stress modules (Hmox1, Gpx1, Cxcl1, Tslp), with the most pronounced corrections in the FB2-FB3 transitional continuum (**Figure 4B**). Histological readouts mirrored these molecular effects: Picrosirius red polarization revealed UV-induced collagen disorganization that was partially restored by ruxolitinib (**Supplementary Figures 4A, B**). Consistently, pathway analysis in FB2-FB3 demonstrated a coordinated reversal under treatment: attenuation of DNA damage, oxidative stress, apoptosis, and senescence, alongside reinforcement of wound-healing and ECM/collagen remodeling pathways (**Figure 4C**).

Pseudotime inference further resolved these shifts at single-cell resolution. In controls, fibroblasts occupy a continuous early-to-late differentiation spectrum with balanced intermediate states (**Figure 4D**). UV irradiation biased this landscape toward late pseudotime and depleted intermediates, consistent with accelerated terminal differentiation. Ruxolitinib re-balanced the trajectory, repopulating early and intermediate states and contracting the late terminal zone, indicative of a directional correction toward homeostasis. Together, these data position JAK1/2 inhibition as a regulator that recalibrates fibroblast dynamics from an “overdriven” differentiation program to a steadier, tissue-maintenance state, predominantly via FB2-FB3.

At the regulatory level, subtype-resolved cadre, most mapping (DoRothEA-anchored) showed a focused rescue centered on transitional fibroblasts (FB1-FB3) (**Figure 4E**; **Supplementary Figure 4C**). The Rux-UV contrast selectively boosted an immediate-early/immune-modulatory cadre, most prominently Nr4a1, with additional gains in Relb, Atf4, Bhlhe40, Egr1, Klf13, Klf9, Mafb, Xbp1, and Zbtb11. In parallel, factors that were UV-favored were counter-shifted under Rux, including Elf1, Esr1, Foxo1, Smad1, and Tead1. Collectively, these patterns depict coherent transcriptional rebalancing that amplifies nodes centered on Nr4a1 and Relb while constraining Foxo/Smad/Tead axes, consistent with broader gene set rescue and improved tissue architecture under ruxolitinib.

Guided by the rescued transcriptional signatures, we posited coordinated epidermal-dermal repair. Ligand-receptor analysis confirmed restoration of basement-membrane signaling from transitional/upper-dermal fibroblasts to keratinocytes under ruxolitinib: Col4a1/Col4a2→Sdc4/Sdc1 interactions were reinstated toward basal and suprabasal layers, canonical cues that organize adhesion complexes and promote re-epithelialization, thereby supporting DEJ integrity under stress (**Figure 4F**). In parallel, fibroblast-to-keratinocyte adhesion inputs mediated by fibronectin/laminin were re-engaged via CD44 and αvβ1 (Cd44, Itgav/Itgb1), while UV-gained, αvβ8-biased collagen/fibronectin signaling (Itgav/Itgb8) was selectively dampened, which is an axis linked to matrix tension and latent TGF-β activation and is consistent with reduced pro-fibrotic tone and DEJ stabilization. Complementing these epithelial-mesenchymal cues, ruxolitinib amplified ANXA1→FPR signaling from FB4 to neutrophils (**Supplementary Figure 4D**), a pro-resolution pathway that curtails neutrophil persistence and facilitates clearance. These intercellular changes provide a mechanistic bridge from transcriptional rescue to the observed anti-inflammatory and matrix-stabilizing phenotypes in vivo and cohere with the histologic improvement in collagen organization.

## Discussion

Using a chronic UVA+UVB induced mouse model, we defined the cellular and transcriptional architecture of photoaged skin at single-cell resolution. Across 14 lineages, UV exposure triggered concerted reprogramming characterized by induction of chronic inflammatory and oxidative-stress modules and broad repression of developmental and differentiation programs, with fibroblasts and keratinocytes emerging as the principal effectors. These features align with the concept of inflammaging, wherein persistent, low-grade inflammation superimposes on intrinsic aging to accelerate tissue decline.

Reclustering resolved eight fibroblast subtypes and clarified their in-situ dynamics. A transitional FB2/FB3 population along the papillary-to-reticular trajectory showed marked amplification of stress/inflammatory signaling and ECM disassembly after UV, whereas papillary subsets (e.g., FB6/FB5) exhibited global transcriptional attenuation. Pseudotime analysis revealed a UV-biased drift toward late, terminal states with depletion of intermediates, consistent with premature differentiation. Together, these observations support a layered model in which early functional suppression of the superficial papillary dermis is followed by inflammation-driven matrix breakdown within transitional fibroblasts, precipitating pathological remodeling in the deeper reticular dermis. Prior single-cell studies in UV-exposed murine skin likewise nominate fibroblasts as dominant inflammatory responders, reinforcing this interpretation.

Given the prominent enrichment of JAK-STAT signaling, most notably in fibroblasts, we asked whether pathway blockades could mitigate photoaging. Topical ruxolitinib (a JAK1/2 inhibitor) has established efficacy in inflammatory dermatoses, where it reduces pruritus and epidermal hyperplasia by dampening Th2/Th22 signaling (e.g., IL-4/IL-13, IL-31). Clinically, ruxolitinib cream was also approved for vitiligo, and combination with narrow-band UVB enhances facial and whole-body repigmentation in multicenter cohorts, mechanistically linked to suppression of the IFN-γ→JAK1/2→STAT1 axis and downstream chemokines CXCL9/10 (16). Consistently, animal and human skin explant studies show that ruxolitinib down-tunes JAK-STAT-driven Th2/IFN inflammation and alleviates epidermal thickening and itch (14). Whether these anti-inflammatory properties extend to structural rescue in photoaging has not been systematically tested.

At the gene and pathway levels, ruxolitinib broadly dampened inflammatory/stress and JAK-STAT-linked modules (e.g., Irf1, Il6, Hmox1, Atf3) while restoring basement-membrane/ECM and adhesion signatures (e.g., Col4a1/Col4a2, Itga5, Thbs1) across fibroblasts and keratinocytes. Shared-rescue analysis highlighted two cross-lineage axes, a stress-metabolic axis (Hmox1, Gpx3, Ucp2, Nr4a2) and a structural-matrix axis (Dcn, Gsn, Nup210l), both of which were directionally reversed by treatment. Cross-reference to GenAge further indicated reversal of canonical aging/stress nodes (Cdkn1a/p21, Trp53, Mif, Txn1, Clock, Insr) in FB and KB/KS, consistent with a systems-level shift from a pro-inflammatory/degradative state toward repair and remodeling.

Mechanistically, a transcription-factor pivot underlies network reprogramming. UV engages a JAK-STAT/IRF/AP-1 inflammatory axis in fibroblasts and keratinocytes, whereas ruxolitinib suppresses this axis and elevates an immediate-early/remodeling program dominated by Nr4a1 (broadest rescue across subtypes) with coordinated gains in Relb, Atf4, Bhlhe40, Egr1, Klf13, Klf9, Mafb, Xbp1, and Zbtb11. In parallel, UV-favored regulators are counter-shifted under ruxolitinib, including Elf1, Esr1, Foxo1, Smad1, and Tead1. The most pronounced TF shifts localized to FB2/FB3 and coincided with restoration of ECM and DEJ programs, reinforcement of wound-healing signatures, and a pseudotime reversion from accelerated terminalization toward homeostatic differentiation. These findings nominate FB2/FB3 as a critical effector subset linking papillary-dermal/DEJ homeostasis to reticular remodeling-that is, the node that executes the stress-to-remodeling transcriptional switch.

Ligand-receptor inference further suggests that ruxolitinib reconstitutes dermal-epidermal crosstalk in a manner compatible with DEJ stabilization: restoration of collagen IV → syndecan and fibronectin/laminin→CD44/αvβ1 cues from fibroblast subsets to keratinocytes, with selective dampening of UV-gained, αvβ8-biased collagen/fibronectin inputs that reinforce matrix stress and TGF-β tone. This signaling pattern coheres with histological improvement and provides a mechanistic bridge between TF-level rewiring and tissue-level repair, consistent with literature assigning these adhesion axes central roles in re-epithelialization and DEJ integrity.

Collectively, our single-cell analysis closes the loop between a disease driver (JAK-STAT/IRF-AP-1) and an actionable intervention (JAK1/2 inhibition) and focuses the response on discrete fibroblast subpopulations, particularly FB2/FB3. These insights motivate two translational strategies: (i) subset-directed regeneration and ECM rebuilding that target FB2/FB3 to couple anti-inflammatory/anti-stress therapy with basement-membrane and collagen restoration; and (ii) spatiotemporally staged interventions that front-load suppression of inflammatory/stress programs and subsequently promote DEJ/ECM reconstruction to favor stable, low-scar repair.

### Limitations

Limitations include the inferential nature of TF activity and cell-cell signaling. We also did not directly assess promoter or enhancer occupancy of collagen-synthesis genes by JAK-STAT factors, so direct transcriptional regulation remains to be established experimentally. Establishing causality for axes such as TWIST2/NR4A/Notch will require genetic perturbation (overexpression/knockdown/knockout) with multi-level functional assays. Ligand-receptor predictions warrant validation by spatial co-localization and orthogonal biophysical readouts. Future ChIP-seq, CUT&RUN, CUT&Tag, or reporter assays will be helpful to explore regulation of collagen synthesis. Species and modeling differences argue for replication in human 3D skin or organoids with spatial transcriptomics and multi-omics (scATAC-seq, proteomics). Finally, translational candidacy will depend on genetic/pharmacologic validation and longer-term efficacy and safety studies.

## Method

### Animals and study design

Female C57BL/6J mice (8 weeks) purchased from Charles River Laboratories (Beijing, China), were housed under specific pathogen-free conditions (12-h light/dark, 22 ± 2 °C, ad libitum chow/water). Mice were randomized to Control (Con), UV, or UV + ruxolitinib (Rux) groups. Sample size per assay is indicated in figure legends; biological replicates refer to independent mice. All procedures complied with institutional and national guidelines and were approved by the Army Medical University ethics committee.

### Construction of photoaging model

Cutaneous photoaging was performed as described previously (17). Briefly, mice were irradiated with a mixed source of UVA (315 nm~400nm, 0.60 mW/cm2) and UVB (290nm~315nm, 3.5 mW/cm2) ray every other day for 12 weeks adapted from our previously validated murine protocol, which reproducibly generates chronic photoaging phenotypes in mice; this combined exposure was selected to model the joint contribution of UVA and UVB to photoaging in a controlled experimental setting (**Supplementary Figure 1A**) (17).

### Topical ruxolitinib intervention

The dorsal skin of mice was treated immediately following each irradiation with vehicle (10% DMSO in Aquaphor) containing ruxolitinib (JAK1/2 inhibitor) (MCE, INCB18424), initially dissolved in DMSO and then mixed in Aquasum (Aquaphor), to achieve 1.5% JAK inhibitor ointment.

### Tissue collection and processing

Twenty-four hours after the final exposure, full-thickness dorsal skin was excised. For histology, tissues were fixed with 4% paraformaldehyde (PFA) and sectioned at 5 µm. For RNA, parallel pieces were snap-frozen in liquid nitrogen. For single-cell work, fresh tissue was processed immediately (below).

### Histological analysis

Hematoxylin and eosin, Masson Trichrome, Picrosirius Red and Elastica-Van Gieson staining were performed using routine procedures. Epidermal thickness and dermal cellularity were quantified from calibrated micrographs. Picrosirius Red was imaged under crossed polarizers (identical illumination/camera settings across groups). Metrics included total birefringent area, orientation coherency, and Type I/III-biased hue distributions, derived via established PSR-polarization protocols. Elastica-Van Gieson’s readouts included elastic fiber area fraction, fragmentation index (endpoints/mm after skeletonization), mean segment length, and tortuosity. All stains were imaged with fixed exposure and background balance. Color deconvolution, segmentation, and morphometry were performed in ImageJ as previously described.

### Immunohistochemistry

Paraffin sections were deparaffinized and underwent heat-induced epitope retrieval (10 mM citrate, pH 6.0, or Tris-EDTA, pH 9.0, per antibody datasheet). Endogenous peroxidase was quenched (3% H_2_O_2_). Sections were blocked (serum/protein block) and incubated with primary antibodies against p16^INK4a (Abcam, ab241543, 1:50), p21 (Cell Signaling Technology #2947, 1:200), and Ki-67 (Abcam, ab15580, 1:200), followed by HRP-linked secondary antibodies and DAB chromogen; nuclei were counterstained with hematoxylin. Negative controls (isotype and secondary-only) and positive control tissues were included in each run. Quantification used automated nuclear segmentation; labeling indices were reported for epidermal basal layer (normalized per mm basement-membrane length) and for dermal compartments.

### Real time quantitative PCR

Total RNA was isolated from fresh skin tissue and cultured cells using TRIzol. Complementary DNA (cDNA) was synthesized with the PrimeScript™ RT Reagent Kit with gDNA Eraser (Perfect Real Time; Takara, Japan). Quantitative PCR (qPCR) was performed with Power SYBR™ Green PCR Master Mix (Applied Biosystems, USA) following the manufacturers’ instructions. β-Tubulin served as the internal reference gene, and relative transcript abundance was calculated by the 2^−ΔΔCt method. All primers were synthesized by Tsingke Biotechnology (Beijing, China).

### Single-cell sequencing

Droplet-based single-cell RNA sequencing was performed on the Chromium platform (10x Genomics) using the Chromium Next GEM Single Cell 3′ Kit v3.1 (PN 1000268) to generate gene-expression (GEX) libraries. Single-cell suspensions were combined with barcoded gel beads and RT master mix, loaded onto a Chromium Next GEM Chip G with Dual Index Kit TT Set A, and processed on a Chromium Controller. After reverse transcription, emulsions were broken and cDNA was amplified to construct GEX libraries. Library yield was quantified with a Qubit 3.0 fluorometer (Life Technologies, 15387293), and fragment size distribution was evaluated using HS DNA chips on a 2100 Bioanalyzer (Agilent, G2939BA). Pooled libraries were sequenced on a NovaSeq 6000 (Illumina) using massively parallel sequencing.

### Single-cell analysis

Analysis was performed as previously described (18). Briefly Raw single-cell RNA-seq reads were processed with Cell Ranger v6.1.2 (10x Genomics) against the mm10 reference genome. Downstream analysis and visualization were performed in Seurat v5.3.0 with standard pipeline and clusters were annotated by canonical marker genes (19, 20). Differential expressions between conditions were assessed using the nonparametric Wilcoxon rank-sum test. Pseudotime were inferred with Monocle 3 (21). Transcription-factor activity was estimated with DoRothEA regulons (22). Ligand-receptor signaling networks were inferred with CellChat v2.2.0 (6). Pathway and Systems Biology Analysis were performed with fgsea v1.22.0 using MSigDB v7.5.1 (23, 24). KEGG pathway enrichment was conducted with clusterProfiler v4.4.4 (25, 26).

### Statistics

Data are presented as mean ± SE. All analyses were conducted in R v4.5.1 (27). Between-group differences were tested with two-tailed unpaired t tests. Statistical significance was defined as P < 0.05; ns, P ≥ 0.05; *P < 0.05; **P < 0.01; ***P < 0.001; **P < 0.0001.

## Data Availability

The dataset have been deposited in GEO under accession number GSE334230.
