# Supplementary material for: Single-cell atlas of photoaged skin reveals JAK-STAT blockade as a strategy to reverse dermal remodeling
Source: Front Immunol. 2026 Jul 1;17:1748123. doi: 10.3389/fimmu.2026.1748123 (PMC13368500; doi:10.3389/fimmu.2026.1748123)
Supplement: Supplementary file 1 [file DataSheet1.docx]

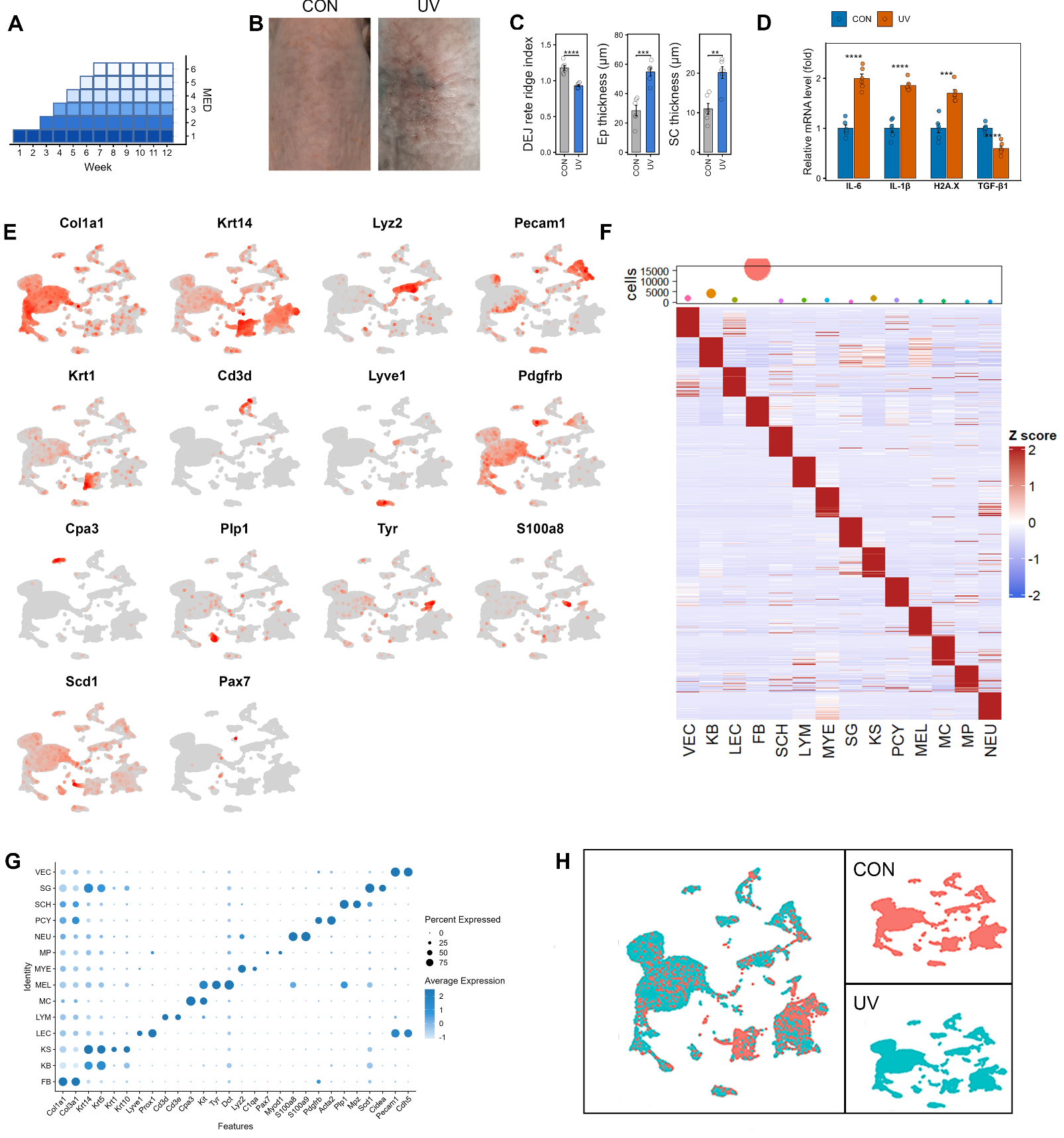


Figure S1. Extended characterization of the murine photoaging model

1. Experimental schematic of the UVA+UVB irradiation regimen used to induce cutaneous photoaging.
2. Representative dorsal skin photographs illustrating macroscopic phenotypes after chronic UV exposure, including pronounced wrinkling (deep furrows) and diminished recoil.
3. Quantitative morphometry: epidermal thickness, stratum corneum thickness, dermal-epidermal junction (DEJ) undulation index. Bars show mean ± SE (n = 6). Statistical significance: ns ≥ 0.05, * < 0.05, ** < 0.01, *** < 0.001, **** < 0.0001.
4. qPCR analysis of UV-injury and senescence-associated transcripts in whole skin. Bar graphs (mean ± SE, n = 6/group) show Il6, Il1β, H2ax, and Tgfb1 in UV versus control (CON). Statistical significance: *** < 0.001, **** < 0.0001.
5. Dot-plot of canonical marker genes across annotated cell types; dot size indicates the fraction of cells expressing each gene, and color intensity reflects average expression. Fibroblast (FB), basal keratinocyte (KB), suprabasal keratinocyte (KS), lymphatic endothelial cell (LEC), vascular endothelial cell (VEC), pericyte (PCY), sebaceous gland (SG), melanocyte (MEL), mast cell (MC), lymphocyte (LYM), myeloid cell (MYE), Schwann cell (SCH), myogenic progenitor (MP), and neutrophil (NEU)
6. Heatmap showing gene expression signatures of each cell type. Each column represents one cell type, and each row indicates the expression of one gene; the size of the dot represents cell number and the value for each gene is row-scaled Z score.
7. UMAP feature maps highlighting exemplar markers per lineage.
8. UMAP embeddings stratified by condition.


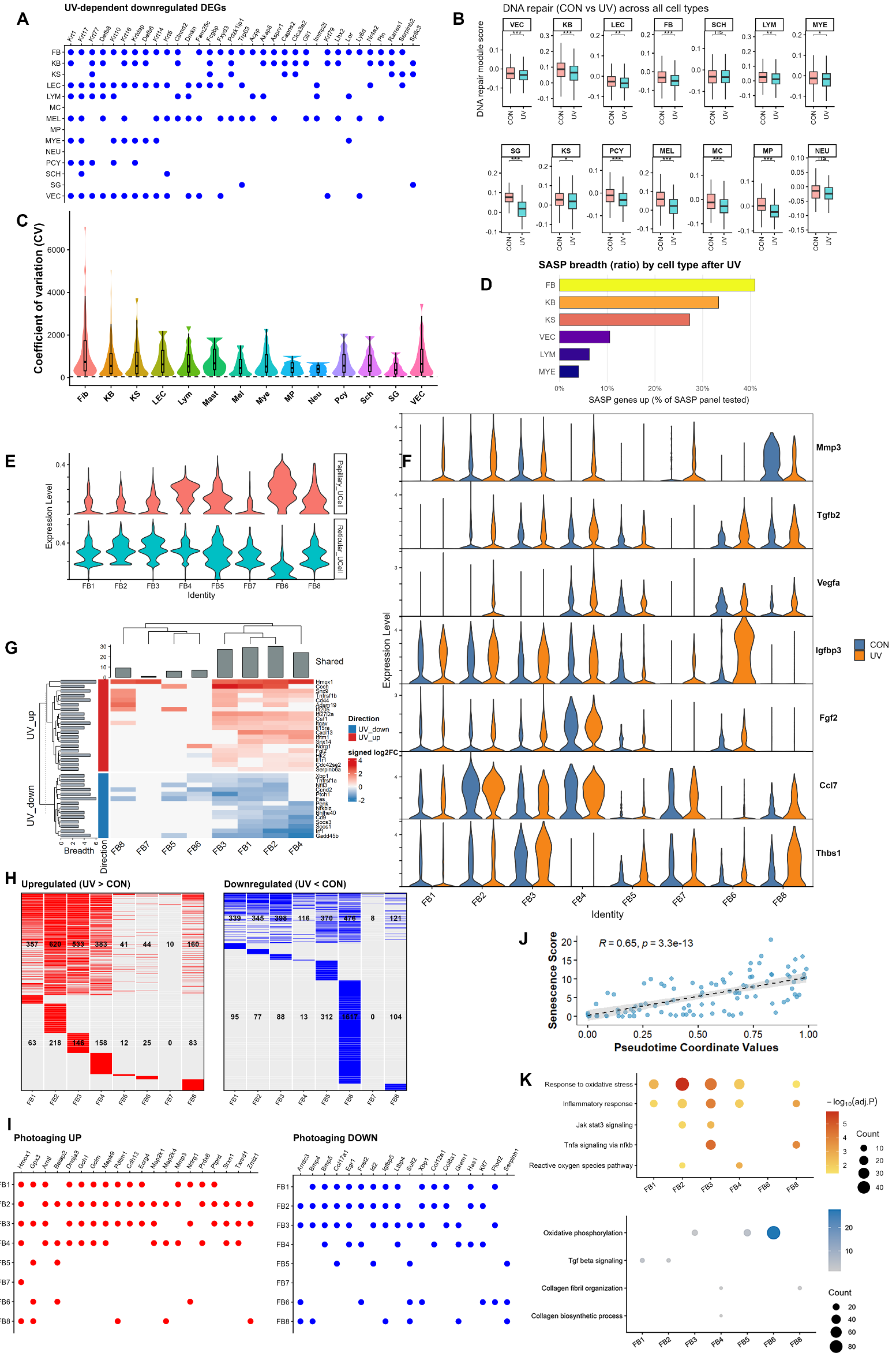


Figure S2. Expanded single-cell profiling of fibroblast states in UV-induced photoaging

1. Cross-lineage convergence of photoaging signatures. Dot plots highlight representative shared DEGs. UV-downregulated genes present in ≥3 lineages;
2. DNA repair program activity across major skin cell classes. Boxplots depict z-scored module scores for a curated DNA damage–repair gene set per cell type; ns ≥ 0.05, * < 0.05, ** < 0.01, *** < 0.001, **** < 0.0001
3. Transcriptional variability by lineage. Violin plots of within-cluster gene-expression dispersion (coefficient of variation, CV).
4. Senescence-associated secretory phenotype (SASP) engagement. Fraction of SASP-panel genes significantly up-regulated by UV in each lineage, indicating differential contribution to inflammatory remodeling.
5. Dermal compartment identities within fibroblast subsets. Violin plots display expression of papillary- versus reticular-dermis signature genes across FB1–FB8 clusters.
6. SASP gene induction in fibroblasts. Violin plots show representative SASP transcripts in Control (CON; blue) versus UV (orange).
7. JAK–STAT signaling in fibroblasts. Heatmap of differential expression between CON and UV; red indicates higher expression in UV, blue indicates lower expression relative to CON.
8. Heatmap partitions UV-induced DEGs into up-regulated (left, red) and down-regulated (right, blue) modules for each fibroblast type (columns); grey, non-significant. The upper tier (above dotted line) denotes DEGs shared by ≥2 lineages, whereas the lower tier captures lineage-restricted changes.
9. Convergent photoaging signatures. Dot plots highlight genes consistently up-regulated (left) or down-regulated (right) by UV in ≥3 fibroblast subsets.
10. Scatter plot illustrating the relationship between Senescence Scores and Pseudotime Coordinate Values.
11. Fibroblast functional reprogramming. GO-BP enrichment for fibroblast DEGs (left, up-regulated; right, down-regulated); Dot size indicates DEG counts and color intensity denotes enrichment significance.


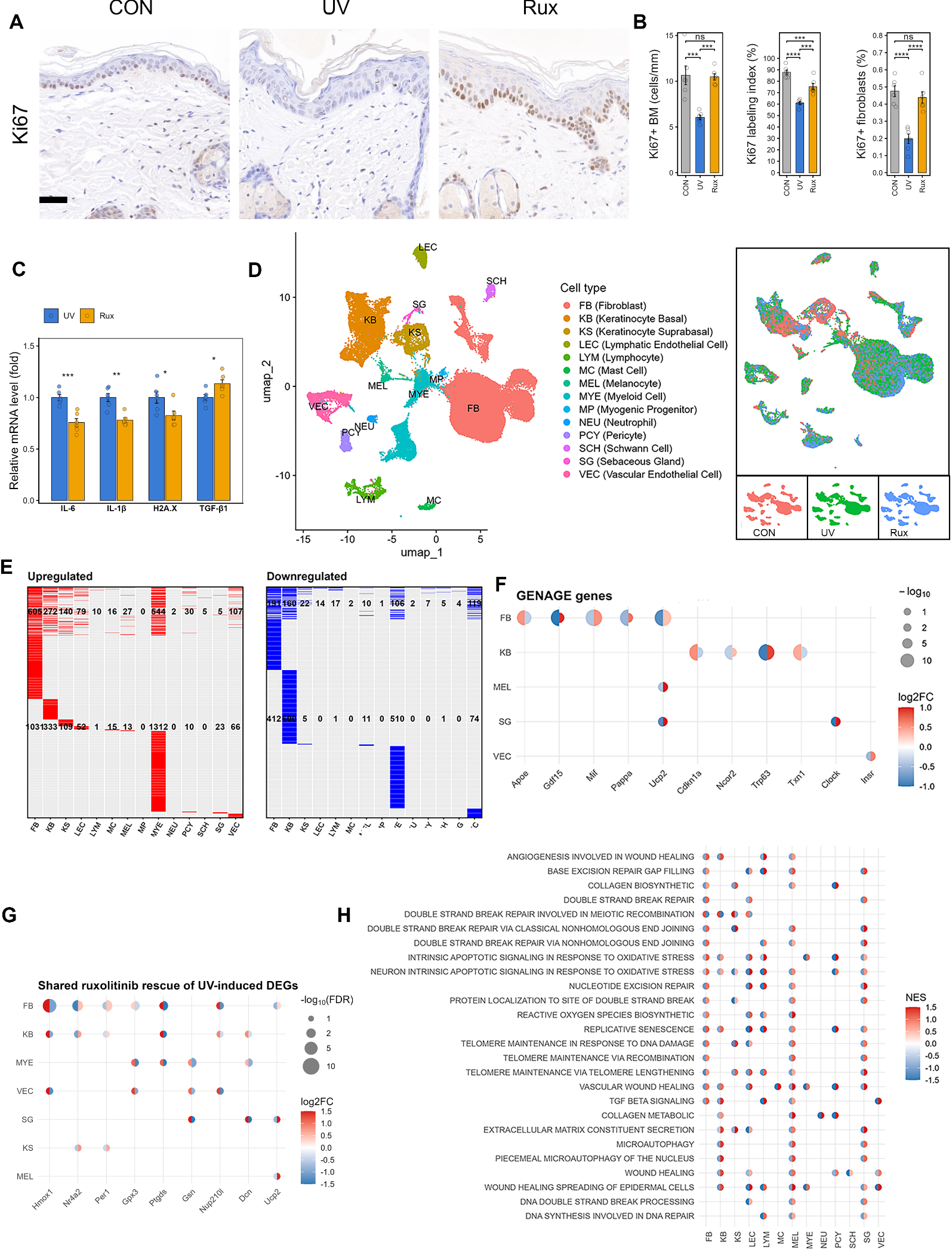


Figure S3. Ruxolitinib mitigates UV-driven DNA damage signals, preserves elastic fiber integrity, and rescues ageing-associated transcriptional programs

1. Ki-67 immunostaining per group (scale bar, 50 µm).
2. Quantification of Ki-67^+ cells per mm basement membrane (BM), Labeling index, and Ki-67^+ dermal fibroblasts per mm² (n = 6). Bars show mean ± SE; ns ≥ 0.05; * < 0.05; ** < 0.01; *** < 0.001; **** < 0.0001.
3. qPCR analysis of UV-injury and senescence-associated transcripts in whole skin. Bar graphs (mean ± SE, n = 6/group). Statistical significance: ns ≥ 0.05; * < 0.05; ** < 0.01; *** < 0.001; **** < 0.0001.
4. UMAP of all single cells colored by cell class (left) and by treatment condition (right), showing global state in cellular composition/state across CON, UV, and Rux.
5. Heatmap partitions Rux-induced DEGs into up-regulated (left, red) and down-regulated (right, blue) modules for each cell type (columns); grey, non-significant. The upper tier (above dotted line) denotes DEGs shared by ≥2 lineages, whereas the lower tier captures lineage-restricted changes.
6. GenAge ageing-associated genes rescued by ruxolitinib across cell types. Each dot denotes a DEG whose UV-induced change is directionally reversed in Rux; left semicircle encodes CON vs UV (red, up in UV; blue, down in UV), right semicircle encodes UV vs Rux (red, increased with Rux; blue, decreased with Rux). Dot size reflects significance.
7. Rux rescue at gene level. Dot plot of rescued genes: left semicircle encodes log-fold change in UV versus CON (red, up in UV; blue, down in UV); right semicircle encodes change between Rux group and UV group (red, up with Rux; blue, down with Rux). Dot size reflects statistical significance.
8. Pathway-level rescue. Dot plot of GO biological processes whose enrichment direction in CON versus UV is reversed by ruxolitinib in the corresponding UV versus Rux contrast. Left semicircle: CON vs UV (red, pathway elevated in UV; blue, reduced in UV). Right semicircle: UV vs Rux (red, increased with Rux; blue, decreased with Rux).


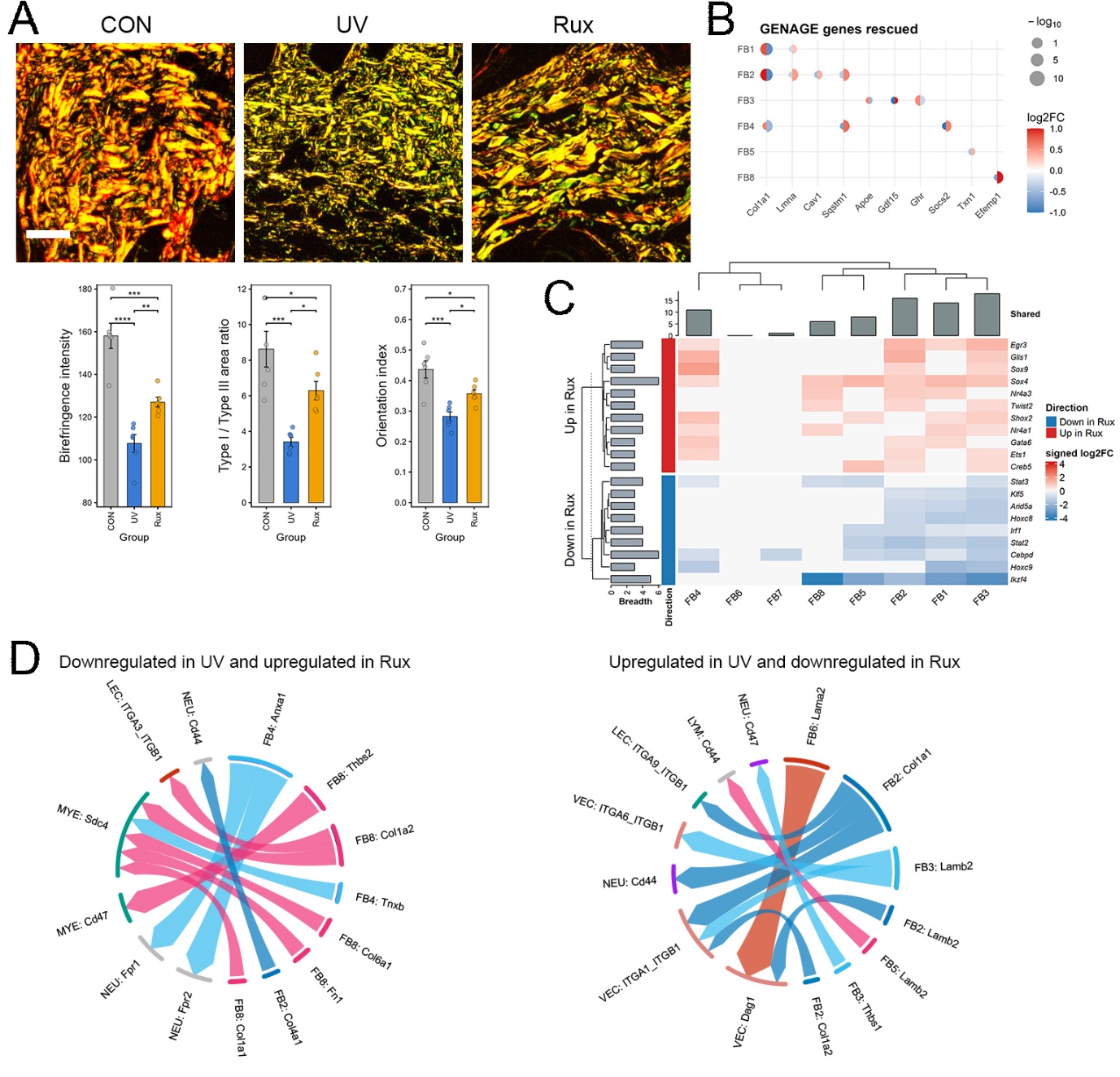


Figure S4. Ruxolitinib modulates pro-inflammatory signaling and restores collagen architecture in UV-photoaged skin

1. Collagen organization by Picrosirius Red (polarized light). Representative micrograph (scale bar, 50 µm) with quantitative readouts below: type I: type III collagen area ratio, birefringence intensity, and orientation coherency. Bars indicate mean ± SE (n = 6 per group). ns ≥ 0.05; * < 0.05; ** < 0.01; *** < 0.001; **** < 0.0001.
2. GenAge ageing genes rescued in fibroblasts. Dot plot of DEGs whose UV-induced shift (CON vs UV) is directionally reversed by ruxolitinib (UV vs Rux). Left semicircle encodes CON vs UV (red, up in UV; blue, down in UV); right semicircle encodes UV vs Rux (red, increased with Rux; blue, decreased with Rux). Dot size reflects significance (adjusted *p*).
3. Transcriptional regulators altered by ruxolitinib. Heatmap of differentially expressed transcription factors between Rux and UV fibroblasts, highlighting attenuation of stress/inflammatory drivers and reinforcement of matrix-remodeling programs.
4. Intercellular communication rescue. Circos map of ligand–receptor interactions between fibroblasts and vascular endothelial cells (VEC), lymphatic endothelial cells (LEC), lymphocytes (LYM), myeloid cells (MYE), and neutrophils (NEU). Links denote interactions perturbed by UV and shifted toward control levels with ruxolitinib; chord thickness indicates interaction strength.
